# RNA and Oxidative Stress in Alzheimer's Disease: Focus on microRNAs

**DOI:** 10.1155/2020/2638130

**Published:** 2020-11-30

**Authors:** Akihiko Nunomura, George Perry

**Affiliations:** ^1^Department of Psychiatry, Jikei University School of Medicine, Tokyo, Japan; ^2^Department of Biology and Neurosciences Institute, University of Texas at San Antonio, San Antonio, USA

## Abstract

Oxidative stress (OS) is one of the major pathomechanisms of Alzheimer's disease (AD), which is closely associated with other key events in neurodegeneration such as mitochondrial dysfunction, inflammation, metal dysregulation, and protein misfolding. Oxidized RNAs are identified in brains of AD patients at the prodromal stage. Indeed, oxidized mRNA, rRNA, and tRNA lead to retarded or aberrant protein synthesis. OS interferes with not only these translational machineries but also regulatory mechanisms of noncoding RNAs, especially microRNAs (miRNAs). MiRNAs can be oxidized, which causes misrecognizing target mRNAs. Moreover, OS affects the expression of multiple miRNAs, and conversely, miRNAs regulate many genes involved in the OS response. Intriguingly, several miRNAs embedded in upstream regulators or downstream targets of OS are involved also in neurodegenerative pathways in AD. Specifically, seven upregulated miRNAs (miR-125b, miR-146a, miR-200c, miR-26b, miR-30e, miR-34a, miR-34c) and three downregulated miRNAs (miR-107, miR-210, miR-485), all of which are associated with OS, are found in vulnerable brain regions of AD at the prodromal stage. Growing evidence suggests that altered miRNAs may serve as targets for developing diagnostic or therapeutic tools for early-stage AD. Focusing on a neuroprotective transcriptional repressor, REST, and the concept of hormesis that are relevant to the OS response may provide clues to help us understand the role of the miRNA system in cellular and organismal adaptive mechanisms to OS.

## 1. Introduction

The process of neurodegeneration in Alzheimer's disease (AD), the most common cause of dementia and a major concern in the aging population across the world, is a dynamic, multifaceted biochemical phenomenon and is essentially lifelong. Oxidative stress (OS) is considered as one of the major underlying mechanisms of AD and related neurodegenerative disorders. Indeed, oxidative damage is closely associated with other pathological key events in neurodegeneration such as mitochondrial dysfunction, inflammation, impaired calcium homeostasis, metal dysregulation, protein misfolding, and impaired autophagy [1, 2]. Several kinds of research materials, i.e., postmortem brain samples and biological fluid from the patients with prodromal-stage of AD, genetically modified animal models of AD, as well as disease models using induced pluripotent stem cells (iPSCs), all provide consistent evidence that OS is a significant early event in the pathological cascade of AD [3–7]. Strikingly, both transgenic animals and iPSC models of AD indicate that a prominent elevation of OS markers occurs simultaneously with or even prior to the initial AD-related amyloid-*β* (A*β*) and tau pathology [5–7].

In contrast to DNA, oxidative damage to RNA has not been a major focus of research until recently, which is presumably due to the assumed transient nature of RNA. However, RNAs including messenger RNA (mRNA) can persist for several hours to days in certain tissues, suggesting that damaged mRNAs are detrimental to the cell if not corrected [8–10]. Increased levels of an oxidized base and an oxidized nucleoside of RNA, 8-oxo-guanine (8-oxoGua), and 8-oxo-guanosine (8-oxoGuo) were demonstrated in the vulnerable neuronal populations in postmortem brains of patients with preclinical AD and mild cognitive impairment (MCI) stage of AD [11–13] as well as in brains of gene-driven animal models of AD at an early stage of degeneration [14, 15]. The 8-oxoGua formation in neuronal RNA is not merely an epiphenomenon but a potentially lethal insult for cells because a recent experimental study elucidates the distinct pathway between heavily oxidized mRNA and apoptotic cell death [16].

Oxidative RNA damage affects not only mRNAs but also noncoding RNA species. Among them, microRNAs (miRNAs), which interfere with the translation of target mRNAs, are of particular interest since their dysregulation has been implicated in neurodegenerative disorders like AD [17, 18]. Indeed, a cellular experiment has demonstrated that miRNAs can be directly oxidized and subsequently misrecognize mRNAs that are not their native targets [19]. Given the notion that noncoding RNAs are expected to contribute towards the biological complexity of the mammalian brain and cognitive evolution [20, 21], oxidative damage to noncoding RNAs and consequent dysregulation of the gene expression might be involved in the central pathophysiology of diverse neuropsychiatric disorders affecting higher brain functions [22, 23]. Besides direct oxidation of miRNAs, there is growing knowledge that OS affects expression levels of multiple miRNAs; conversely, miRNAs regulate lots of genes involved in the OS response [24, 25]. Intriguingly, several miRNAs embedded in oxidative stress regulation are involved also in several known pathways of neurodegeneration in AD, Parkinson's disease (PD), amyotrophic lateral sclerosis, and Huntington's disease [26, 27]. Further investigations towards elucidating the possible involvement of the oxidatively modified miRNAs and oxidatively altered miRNAs networks in the neurodegenerative pathways may open a novel avenue to establish an early intervention strategy for AD.

## 2. Direct Oxidation of RNA Species

### 2.1. Susceptibility of RNA to Oxidative Damage

Compared to oxidative DNA damage, far fewer studies have focused on oxidative damage to RNA, and only limited kinds of oxidatively modified bases in RNA have been reported previously [28–30]. Among multiple adducts of nucleoside oxidation, 8-oxo-deoxyguanosine (8-oxodGuo) and 8-oxoGuo are two of the best characterized and studied forms of DNA and RNA oxidation, respectively [31, 32]. Cellular abundance, location, lack of coating histone, and its single-stranded structure make RNA more susceptible to OS than DNA [33–35] (Table 1). Accordingly, greater oxidation to RNA than to DNA has been shown in studies with isolated DNA and RNA as well as in several kinds of cell lines and tissues [8].

### 2.2. Susceptibility to Oxidative Damage in Different RNA Species

#### 2.2.1. Messenger RNA (mRNA)

The extent of oxidative RNA damage varies greatly among different types of RNA in a manner that appears to show greater oxidative RNA damage in lesser association with RNA binding proteins [10]. For example, poly (A)^+^ mRNA is found to have fivefold higher levels of 8-oxoGuo than total RNA that consists mostly of ribosomal RNA (rRNA) [9]. In the AD brain, northwestern blotting with a monoclonal anti-8-oxoGuo antibody shows that a significant amount of brain poly (A)^+^ mRNA species are oxidized. While the identified oxidized mRNAs reveal that some species are more susceptible to OS in AD, no common motifs or structures were found in the oxidatively susceptible mRNA species in the AD brain [36]. Some of the identified known oxidized transcripts are related to the pathophysiology of AD, which included p21ras, mitogen-activated protein kinase (MAPK) kinase 1, carbonyl reductase, copper/zinc superoxide dismutase (SOD1), apolipoprotein D, and calpains, but not amyloid-*β* protein precursor (APP) or tau [36].

#### 2.2.2. Ribosomal RNA (rRNA) and Transfer RNA (tRNA)

In the AD brain, rRNA, extremely abundant in neurons, contains 8-oxoGuo [37, 38]. Remarkably, rRNA shows higher binding capacity to redox-active iron than tRNA, and consequently, the oxidation of rRNA by the Fenton chemistry forms 13 times more 8-oxoGuo than tRNA [37].

#### 2.2.3. MicroRNA (miRNA)

MicroRNAs (miRNAs) are small noncoding RNAs containing approximately 22 nucleotides, and they bind to complementary sequences in the three prime untranslated regions (3′UTRs) of target mRNA transcripts, thereby inhibiting mRNA translation or promoting mRNA degradation [39]. In the biogenesis pathway, miRNAs are transcribed from genomic loci as long as primary strands (pri-miRNAs) that undergo cleavage to precursor hairpins (premiRNAs) in the nucleus before being exported to the cytoplasm. PremiRNAs are further cleaved into double-stranded miRNA : miRNA∗ duplexes and loaded into the RNA-induced silencing complex (RISC) containing an Argonaute (Ago) family protein as a core component. Finally, single-stranded mature miRNAs are completed by unwinding of the duplexes [40]. As a result, the repressive functions of the RISC and mature miRNAs themselves have been primarily associated with the cytoplasm, while several mature miRNAs are expressed also in the nucleus and nucleolus [41, 42]. Given cytoplasmic predominance of the subcellular localization and the single-stranded form, the mature miRNAs might be highly vulnerable to reactive oxygen species (ROS) (Table 1). Surprisingly, up to hundreds of miRNAs, besides 22 tRNAs and two rRNAs, have been identified from mitochondria, the major source of ROS [41]. Furthermore, miRNAs are often relatively long lived, and their half-lives reported to be ranged from 28 to 220 h, which is roughly 2- to 20-fold longer than that of typical mRNAs (about 10 h) [43]. Taken together, these findings support the possibility that miRNAs have abundant opportunities to be attacked by ROS.

Wang et al. have demonstrated that oxidized miRNAs containing 8-oxoGuo are produced in vitro through hydrogen peroxide (H_2_O_2_) treatment in a rat heart cell line as well as in vivo in the hearts of a mouse model of ischemia/reperfusion [19]. Indeed, miR-184 is the most highly oxidized but other oxidized forms of miRNAs are detected, i.e., oxidized miR-135a, miR-139, miR-204, miR-21, miR-23a, miR-290, miR-29a, and miR-30c. Therefore, not all miRNAs are oxidized in response to ROS stimulation, whereas several miRNAs are more susceptible to the oxidative modification. It remains to be determined whether there exists a common motif and sequence for the selective oxidation of miRNAs [19]. Although oxidized miRNAs are not identified in the human brain diseases at the current moment, possible involvement of the oxidized miRNAs in AD and related neurodegeneration is strongly suggested [8, 44, 45]. Additionally, direct oxidative modifications to other noncoding RNAs such as small nuclear RNAs, small nucleolar RNAs, long noncoding RNAs, and circular RNAs are not reported, while the role of these RNA species in the nervous system has recently drawn growing attention [46, 47].

### 2.3. Sources of Reactive Oxygen Species (ROS) Responsible for RNA Oxidation

#### 2.3.1. ROS of Mitochondrial Origin and Fenton Reaction

The brain is particularly vulnerable to oxidative damage because of its high oxygen consumption rate (accounting for 20-25% of total body oxygen consumption but less than 2% of total body weight), high content of fatty acids and transition metals, and relative paucity of antioxidant enzymes compared with other organs (e.g., the content of catalase in the brain is only 10-20% of the liver and heart) [8, 48] (Table 1). Given this environment, neurons are continuously exposed to ROS such as superoxide (O_2_·^−^), H_2_O_2_, and hydroxyl radical (·OH) that are produced from the mitochondrial electron transport chain through the normal cellular metabolism [49–51]. Among them, highly reactive ·OH can diffuse through the tissue only in the order of several nanometers [52], while O_2_·^−^ is hardly permeable through cell membranes [53]. In consideration of the widespread damage to cytoplasmic RNA in AD [54, 55], RNA species are likely attacked by ·OH, which is formed from the reaction of highly diffusible H_2_O_2_ [56] with redox-active metals through the Fenton chemistry [37]. In the AD brain, malfunctioning mitochondria likely play a central role in producing abundant ROS as well as supplying redox-active iron into the cytosol [57, 58]. Indeed, ribosomes purified from the AD hippocampus contain was significantly higher levels of redox-active iron compared to controls, and the iron is bound to rRNA [37]. Therefore, mitochondrial abnormalities coupled with dysregulation of metal homeostasis are key features closely associated with ROS formation responsible for the RNA oxidation in AD [59].

#### 2.3.2. Mode of Oxidized miRNA Generation

The concept that ROS originates from the mitochondria and becomes harmful when they are coupled with redox-active metals is fully compatible with the mode of the oxidized miRNA generation in the cellular model reported by Wang et al. [19]. In fact, miRNAs remain almost intact after incubation with H_2_O_2_ or redox-active iron (Fe^3+^) alone. However, when miRNAs were incubated in a mixture of H_2_O_2_ and Fe^3+^ along with the reducing agent ascorbate, their oxidation substantially occurred, which indicates that oxidatively modified miRNAs are generated by ·OH via the Fenton reaction [19].

### 2.4. Biological Consequence of Oxidized RNA

#### 2.4.1. Oxidized mRNA

Although guanine is the most reactive of the nucleic acid bases, not only 8-oxoGuo but also 8-oxo-adenosine, 5-hydroxycytidine, and 5-hydroxyuridine have been identified in oxidized RNA [28]. These oxidized nucleosides may have altered pairing capacity and thus be at the origin of erroneous protein production. Indeed, the 8-oxoGua can pair with both adenine and cytosine, and thus oxidized RNA compromises the accuracy of translation [60, 61]. The oxidized bases in mRNAs in cell lines cause ribosomal stalling on the transcripts leading to a decreased rate of full-length peptide synthesis [9, 62, 63], as well as synthesis of truncated, nonfunctional peptides, or mutated peptides [64]. Moreover, strand scission is proposed to result from as many as 40% of reactions of ·OH with RNA [65]. Recently, a distinct pathway between heavily oxidized mRNA and apoptotic cell death has been established. Mechanistically, specific binding of poly(C)-binding protein 1 (PCB1) to oxidized mRNA in which two 8-oxoGua residues are located nearby induces caspase-3 activation and subsequent apoptosis [16].

#### 2.4.2. Oxidized rRNA

The biological consequence of rRNA oxidation has also been investigated in vitro using translation assays with oxidized ribosomes from rabbit reticulocytes and shows a significant reduction of protein synthesis [37]. Notably, studies on brains of subjects with AD and MCI have demonstrated ribosomal dysfunction associated with oxidative RNA damage. Isolated polyribosome complexes from AD and MCI brains show a decreased rate and capability for protein synthesis without alteration in the polyribosome content [38, 66]. A variety of ways that oxidative damage to the functional domain of rRNA which affects translation are suggested: (i) inhibiting ribosomal assembly, resulting in the nonfunctional subunit; (ii) interfering with codon-anticodon interaction or with binding of *aminoacyl*-*tRNA,* causing miscoding or ribosomal stalling, and (iii) blocking elongation of nascent peptide and stalling the ribosome [10].

#### 2.4.3. Oxidized tRNA

Oxidative damage to tRNA may cause defects in codon-anticodon pairing or in aminoacylation, potentially leading to production of miscoded proteins [10]. Moreover, cleavage and fragmentation of tRNA induced by oxidative stress are observed in cell lines [67], which promotes formation of stress granules [10]. Like oxidized mRNA, a mechanism inducing apoptosis has been suggested by oxidized tRNA. Because tRNA is accessible in the mitochondrial intermembrane space, oxidized tRNA forms crosslinked complex with cytochrome *c* (cyt *c*), which facilitates cyt *c* release from the mitochondria and subsequently induces apoptosis [68].

#### 2.4.4. Oxidized miRNA

A recent discovery of oxidized miRNAs opens new avenue in the research field of RNA oxidation. The oxidized miRNA causes misrecognizing target mRNAs, resulting in downregulation in synthesis of particular proteins and potentially inducing cellular crucial events [19]. Among several oxidized miRNAs, oxidized miR-184 containing 8-oxo-Guo shows such a prominent influence to the cell functions. Indeed, oxidized miR-184 associates with the 3′UTRs of B cell lymphoma-extra-large (Bcl-xL) and Bcl-2-like protein 2 (Bcl-w) that are not its native targets. Subsequent reduction of antiapoptotic proteins Bcl-xL and Bcl-w is involved in the initiation of apoptosis in the study with a rat heart cell line. Moreover, the reduction of these proteins by oxidized miR-184 is associated with an increase in susceptibility of the heart to infarction in a mouse model of ischemia/reperfusion injury [19]. It has been identified also that oxidized miR-204, but not native miR-204, can regulate pancreatic and duodenal homeobox 1 C-terminal inhibiting factor 1 (Pcif1), and oxidized miR-139, but not native miR-139, can regulate RNA (guanine-7-) methyltransferase (RNMT), further suggesting that oxidative modification can affect the targets of miRNAs [19]. Given the notion that miRNAs are expected to contribute towards the biological complexity of the mammalian brain and cognitive evolution [20, 21], the elucidation of other oxidized miRNAs and their targets may shed new light on understanding the complex molecular mechanism of brain aging and neurodegenerative diseases.

The mechanisms inducing apoptosis by the oxidized RNAs are summarized in Table 2. Most importantly, RNA oxidation leads to not only impaired RNA normal functions but also the gain of a signal to facilitate cellular apoptosis in response to OS.

### 2.5. Coping with RNA Damage

#### 2.5.1. Degradation

Degradation of RNA plays a central role in RNA metabolism, and damaged RNA can be removed through degradation by ribonucleases (RNase), but selective degradation activity for oxidized RNA has not been established for known RNases [34, 69]. Because absence of no-go decay factors causes an accumulation of 8-oxoGuo-containing mRNA in yeast, the oxidized mRNA is likely subjected to degradation through no-go decay, a ribosome-based mRNA surveillance mechanism [9].

#### 2.5.2. Repair Mechanisms

Alkylation damage in RNA is repaired by the same mechanism as a DNA-repair, catalyzed in the bacterium *Escherichia coli* by the enzyme AlkB, and in humans by the related protein [70]. However, specific repair mechanism for oxidized RNA, unlike DNA, has not been reported.

#### 2.5.3. Avoidance of Oxidized Ribonucleotides Incorporation

The mechanism avoiding incorporation of the oxidized nucleotide into DNA and RNA is involved in coping with nucleic acid damage [33, 34, 71, 72]. MutT homologue 1 (MTH1) and Nudix type 5 (NUDT5) proteins participate in this error-avoiding mechanism by hydrolyzing the oxidized nucleoside diphosphates and/or triphosphates to the monophosphates [60, 61, 73]. Indeed, the expression of MTH1 is increased in vulnerable neurons in AD [74] and PD [75], indicating a compensatory upregulation of the MTH1 against OS. Moreover, several enzymes involved in the nucleotide metabolism show a discriminator activity against the oxidized nucleotides. Guanylate kinase (GK), converting GMP to GDP, is inactive on 8-oxo-GMP [76]. Similarly, ribonucleotide reductase (RNR), catalyzing reduction of four naturally occurring ribonucleoside diphosphates, is inactive on 8-oxo-GDP [76]. The final gatekeeper is RNA polymerase that incorporates 8-oxo-GTP into RNA at a much lower rate compared to the normal GTP incorporation [34, 60, 72].

#### 2.5.4. Proteins Binding Specifically to Oxidized RNA

Proteins that bind specifically to 8-oxoGuo-containing RNA have been reported, namely, polynucleotide phosphorylase (PNPase) [77, 78], Y box-binding protein 1 (YB-1) [79], and AU-rich element RNA binding protein 1 (Auf1) [also called heterogeneous nuclear ribonucleoprotein D0 (HNRNPD)] [80, 81]. It has been proposed that these proteins are able to recognize and discriminate the oxidized RNA molecule from normal ones, thus contributing to the fidelity of translation in cells by sequestrating the damaged RNA from the translational machinery [77–80].

#### 2.5.5. Coping with Oxidative miRNA Damage

Compared to the biogenesis and activity, the process of degradation of miRNAs has received less attention. While miRNAs are globally stable, individual miRNAs display rapid decay dynamics in some specific situations, as occurs for a few neuron-enriched miRNAs, but not constitutively expressed miRNAs, and for mature miRNAs, but not the miRNA precursors [43]. Although several miRNA-degrading enzymes have been identified, including both 3′-to-5′ and 5′-to-3′exoribonucleases [43, 82], whether they are less or more efficient for oxidized miRNAs is unknown. Among them, the human PNPase degrades certain mature miRNAs in human melanoma cells [82] and may be involved in oxidized miRNA degradation [77, 78]. A recent comprehensive review has concluded that how cells cope with oxidative damage to miRNAs is unclear, and whether they have evolved pathways to degrade or repair them should be the subject of future research [83].

### 2.6. Therapeutic Interventions against Oxidized RNA Species

Several experimental studies on human subjects and animal models have demonstrated successful interventions including nonpharmacological and pharmacological approaches towards reduction of oxidized RNA. Indeed, the efficacious interventions are caloric restriction and exercise in human subjects [84], as well as administrations with antioxidants and anti-inflammatory agents such as acetyl-l-carnitine [85], *α*-linolenic acid [86], *α*-lipoic acid [85], docosahexaenoic acid (DHA) [86], indomethacin [87], selenium [14], and vitamin E [88] in experimental animals. Antioxidants may reduce miRNA oxidation as well. Administration of *N*-acetylcysteine (NAC) reduces oxidized miR-184 and attenuates the reductions in Bcl-xL and Bcl-w and consequently prevents apoptosis both in the rat heart cell line and in the hearts of a mouse model of ischemia/reperfusion [19]. Overexpressing human *MTH1* in a transgenic mouse model significantly reduced 8-oxoGuo in the brain [89].

## 3. microRNAs and Oxidative Stress Regulation

### 3.1. Interplay between miRNAs and Oxidative Stress (OS) Affects Neurodegeneration

MiRNAs are embedded in complex regulatory networks, since a single miRNA can regulate up to hundreds of target mRNAs coding for different proteins [39, 90]. It has been demonstrated that miRNAs regulate lots of genes involved in the OS response, and conversely, OS affects expression levels of multiple miRNAs. Indeed, 27 different miRNAs are associated with generation but 11 different miRNAs are associated with reduction of OS. In downstream of OS, induced expression of 56 miRNAs, while reduced expression of 32 miRNAs, is observed [25]. Moreover, dysregulation of miRNAs has been implicated in neurodegenerative disorders such as AD, PD, amyotrophic lateral sclerosis, Huntington's disease, and prion disorders [27, 91]. Although the interrelations among miRNAs, OS, and neurodegeneration are often considered separately, several miRNAs embedded in OS regulation or OS targets are involved also in known pathways of neurodegeneration such as mitochondrial dysfunction, inflammation, and protein misfolding. Some classes of miRNAs *accelerate* these cellular anomalies, whereas others act in a counterregulatory, *protective* role. Also, changes in levels of certain species of miRNAs can be a *consequence* of the abovementioned anomalies [17, 27].

### 3.2. Specific miRNAs Associated with Both OS and Alzheimer's Disease (AD)

#### 3.2.1. Altered miRNAs in the AD Brain at Braak Stages III/IV

The roles of individual miRNAs, i.e., seven upregulated miRNAs (miR-125b, miR-146a, miR-200c, miR-26b, miR-30e, miR-34a, miR-34c) and three downregulated miRNAs (miR-107, miR-210, miR-485) that are relevant to both OS and neurodegenerative pathways in AD are summarized in Table 3. Of note, all the alterations of these miRNAs are demonstrated in the human brain with early AD pathology at Braak stages III and IV [92–96] that are not a substrate for profound cognitive impairment [97] and largely correspond to MCI [98].

#### 3.2.2. Upregulated miRNAs in the AD Brain at Braak Stages III/IV

A synergistic effect mechanism of accelerated AD pathology and OS in neurodegeneration is found in several upregulated miRNAs listed in Table 3, i.e., miR-125b [99, 100], miR-146a [101–103], miR-26b [95], and miR-34a [104–108]. Upregulation of these miRNAs leads to A*β* production and/or tau-phosphorylation and an increase in vulnerability to OS. Upregulation of miR-34c increases ROS production and mediates synaptic deficits through the ROS-Jun amino terminal kinase (JNK)-p53 pathway [109]. In contrast, upregulation of miR-30e, being found in the hippocampus of AD [93], increases antioxidant enzymes such as SOD, glutathione (GSH), and glutathione-peroxidase (GSH-PX) and decreases ROS [110], suggesting compensatory regulation of the miRNA in the AD brain. The role of miR-200c in the pathophysiology is more complicated. MiR-200c is upregulated by A*β*-induced endoplasmic reticulum (ER) stress or OS [111, 112] and able to reduce A*β* secretion [113], suggesting a compensatory role. Indeed, upregulation of miR-200c is observed in the hippocampus of AD subjects [93] and in the cortices of APP transgenic mice at an early period of A*β* deposition [113]. In epilepsy model rats, however, downregulation of miR-200c is neuroprotective. It increases SOD and GSH-PX activities and decreases apoptosis of hippocampal neurons by upregulation of its target reversion-inducing cysteine-rich protein with kazal motifs (RECK) via inactivating protein kinase B (AKT) signaling [114].

#### 3.2.3. Downregulated miRNAs in the AD Brain at Braak Stages III/IV

As for downregulated miRNAs listed in Table 3, each specific miRNA shows its own mode of relevance to the disease pathogenesis. OS triggers downregulation of miR-107 that normally represses A*β* production, and downregulation of miR-107 in AD is associated with an accumulation of A*β* [115, 116]. Because cell-derived soluble A*β* induces further OS [117], a vicious cycle promoting OS and A*β* production is formed. Similarly, miR-485 that normally represses A*β* production and silences OS [118, 119] is downregulated in the cortex of AD [96], where altered miR-485 exacerbates neurodegeneration through both excess of A*β* and OS. Conversely, downregulation of miR-210 in the hippocampus of AD [93] may represent compensatory regulation of the miRNA in the AD brain. Because soluble A*β* leads to upregulation of miR-210 that inhibits the mitochondrial respiration and function [116], downregulation of miR-210 should be neuroprotective through alleviating A*β* toxicity and OS silencing.

#### 3.2.4. Other Altered miRNAs in the AD Brain

Downregulation of miR-128 is observed in the hippocampus at the Braak stage VI of advanced AD patients, but not at the Braak stage III/IV [94]. Indeed, miR-128 promotes A*β*-mediated cytotoxicity by targeting peroxisome proliferator activated receptor-*γ* (PPAR-*γ*) via activation of nuclear transcription factor *κ*B (NF-*κ*B) [120]. Also, miR-128 promotes OS by targeting sirtuin 1 (SIRT1) [121], indicating that downregulation of miR-128 in late-stage AD seems to be a compensatory regulation against A*β* pathology and OS. MiR-15b also downregulated in the temporal cortex and hippocampus of patients with AD. In contrast to miR-128, miR-15b reduces A*β* accumulation through directly targeting 3′UTRs of *β*-site amyloid precursor protein-cleaving enzyme 1 (BACE1) and NF-*κ*B [122] and counteracting senescence associated mitochondrial dysfunction and ROS generation by targeting stress-induced SIRT4 [123]. Therefore, downregulation of miR-15b in AD is associated with an acceleration of both A*β* pathology and OS.

**Figure 1:**
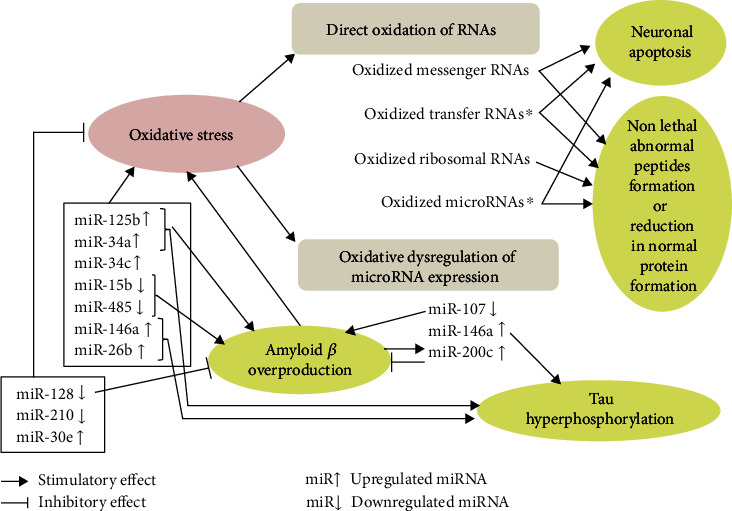
Overview of interrelations among oxidative stress, RNA species modification/dysregulation, and neurodegenerative changes in Alzheimer's disease. Oxidative stress can induce two modes of oxidative insults on RNA species, i.e., direct oxidation of RNAs and oxidative dysregulation of the microRNA (miRNAs) expression. These modifications and dysregulations of RNAs potentially induce neuronal apoptosis or nonlethal neuronal dysfunction as well as amyloid *β* overproduction and tau hyperphosphorylation. Besides these changes as consequences of oxidative stress, altered expressions of some miRNAs are associated with an acceleration of oxidative stress, while those of others are associated with a compensatory reduction of oxidative stress. Of note, dysmetabolism of amyloid *β* can be a cause or a consequence of oxidative stress. ^∗^Oxidized transfer RNAs and oxidized microRNAs have been reported only in cellular and animal models, but the other changes in RNAs shown in this figure have been found in the brains of Alzheimer's disease.

The interrelations among OS, RNA species modification/dysregulation, and neurodegenerative changes in AD are summarized in Figure 1.

#### 3.2.5. Altered miRNAs in the Mouse Model of AD

Downregulation of miR-20a is observed in the transgenic mouse model of AD carrying *Swedish* double mutation (K670 N/M671L) of the amyloid precursor protein (APP) and M146L mutation of the presenilin-1 (PSEN1) (APPswe/PSEN1M146L) [108]. While ROS upregulates miR-20a in primary hippocampal neuron [124], miR-20a reduces A*β* by targeting APP [125], indicating a decrease in the protective function of miR-20a in this model. Conversely, miR-98 is upregulated in the APPSwe/PSEN1M146L mouse model [108], which seems to represent a compensatory mechanism against AD and OS. MiR-98 decreases the production of APP and A*β* and improves OS and mitochondrial dysfunction targeting hairy and enhancer of split- (Hes-) related with the YRPW motif protein 2 (HEY2) via inactivation of the Notch signaling pathway [126]. Similarly, a protective function of miR-330 targeting vav guanine nucleotide exchange factor 1 (VAV1) is reported in the AD mouse model through the MAPK signaling pathway. Upregulation of miR-330 reduces A*β* production and alleviates OS and mitochondrial dysfunction in AD [127]. In the transgenic mouse model of AD carrying APP Swedish mutation and PSEN1 lacking exon 9 (APPswe/PSEN1*Δ*9), OS-associated miR-34a, miR-34c, and miR-98 are abnormally expressed in the animal between 3 and 6 months of age [128] when initial A*β* depositions start in the hippocampus [129]. Taken together, studies on the postmortem human brain and transgenic animal model consistently suggest the altered expression of the OS-associated miRNAs as an early-stage event of AD.

### 3.3. Neuroprotective Function of REST and miRNAs

#### 3.3.1. A Transcriptional Repressor, REST: AD and OS

The restrictive element-1 silencing transcription factor (REST), also known as neuron-restrictive silencing factor (NRSF), has been thought to function as a regulator of neuronal genes involved in neurogenesis and neuronal differentiation. However, recently accumulated findings strongly indicate that REST is not only a classical repressor to maintain normal neurogenesis but it is also a fine fundamental protector against neurodegeneration [130, 131]. Surprisingly, the expression of REST is increased with aging in brains of cognitively normal elderly. However, in brains of patients with MCI and AD, a significant reduction in REST has been found in cortical and hippocampal neurons, where nuclear REST levels are positively correlated with a global cognitive measure. Of particular note, elevated REST levels are associated with preservation of the cognitive function even in the presence of AD pathology [132]. Indeed, chromatin immunoprecipitation with deep sequencing shows that REST represses proapoptotic genes, e.g., FAS, FAS-associated death domain protein (FADD), TNF receptor-associated death domain protein (TRDD), Bcl-2-associated X protein (BAX), and cytochrome *c*, as well as AD pathology-associated genes, e.g., presenilin-2 (PSEN2) and presenilin enhancer 2 (PSENEN) implicated in A*β* generation and MAPK implicated in tau phosphorylation. Simultaneously, REST induces the expression of forkhead box O (FOXO) transcription factors that mediate OS resistance and the antioxidant enzymes catalase and SOD1. That REST potently protects neurons from OS and A*β* toxicity is rigorously confirmed by experiments on conditional REST knockout mice and nematode *Caenorhabditis elegans* (*C. elegans*) models with a functional orthologue of REST and SPR-4 [132].

#### 3.3.2. Rest and miRNAs

As REST has been shown to mediate the temporal and cell-specific expression of several classes of noncoding RNAs including miRNAs [133], the loss of REST in AD might disturb the network of miRNAs. Quite recently, loss of REST causes upregulation of miR-124 and downregulation of its target protein phosphatase 1 (PTPN1) and disrupted miR-124/PTPN1 signaling that induces AD-like tau pathology in mice via activation of glycogen synthase kinase 3 (GSK-3) and inactivation of protein phosphatase 2A (PP2A) [134]. Conversely, REST is regulated by some cytokines/regulators such as miRNAs [130]. Indeed, miR-124 and miR-9 target REST and form self-enforcing loops together with the polypyrimidine tract-binding protein (PTB) to control neuronal conversion and maturation. In a double negative feedback loop consisting of PTB, miR-124, and REST (PTB-REST-miR-124 loop), REST represses miR-124, which in turn dismantles multiple components of the REST complex, and the RNA binding protein PTB serves both as a substrate for and a key inhibitor to the miR-124 targeting [135]. As is the case with the process of neurogenesis, the miRNA system likely acts both upstream and downstream of the REST expression in the aging brain, where the miRNA system participates in fine tuning of the gene to distinguish neuroprotection from neurodegeneration.

### 3.4. MiRNAs Mediates Hormesis under OS

#### 3.4.1. Hormesis and OS

The relationship between the strength of a stressor and its biological effects on a physiological phenotype is not linear, but biphasic. This process is known as hormesis, whereby exposure to a low dose of a potentially harmful stressor promotes adaptive changes to the cells, organs, and living body that enables it to better tolerate subsequent stress. When we consider the hormetic response in association with neuronal adaptation to stressors, it is helpful to introduce the concepts of “neurohormesis” by Mattson [136] and “mitohormesis” by Ristow [137] in the context of maintaining cognitive health. Because these concepts indicate that moderate levels of OS are beneficial to the biological system through the adaptive preconditioning, further investigation to understand the molecular mechanisms of the regulatory hormesis may give hints on developing a new therapeutic approach for neurodegenerative diseases.

#### 3.4.2. Hormesis and miRNAs

**Figure 2:**
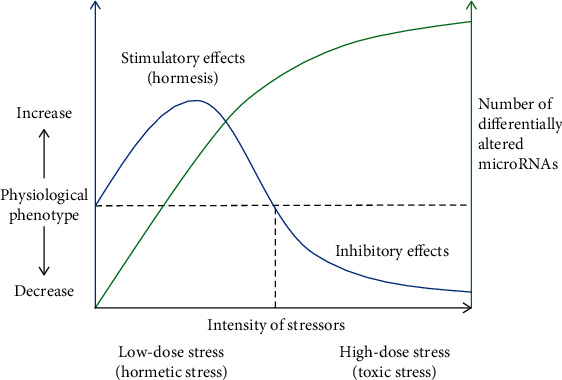
A microRNA system mediates hormesis. As intensity of stressors increases, the number of differentially altered miRNAs increases, which is associated with a biphasic physiological phenotype. Under low doses of hormetic stress, a moderate increase in miRNAs is associated with an increase in physiological (beneficial) phenotype. However, under high doses of toxic stress, an excessive increase in miRNAs is associated with a decrease in physiological phenotype.

Recent studies indicate an involvement of changes in miRNAs in the adaptive response of hormesis or preconditioning. In an experiment using *C. elegans* exposed to nicotine in the period of postembryonic stages, the hormetic response in locomotive speed of the worm was observed by low and high dose of nicotine exposure. Of note, there was a dose-dependent increase in the degree of fold change (1 vs. 3.4-fold) and the number (1.3% vs. 16.4%) of the differentially altered miRNAs in the worm treated with low- and high-dose nicotine [138]. These observations support a speculation that miRNAs as a system mediates hormesis (Figure 2). In another study using *C. elegans*, an antibiotic enoxacin extends the lifespan of the worms by downregulating miR-34-5p. The longevity effects associated with downregulated miR-34 are abrogated by the antioxidant NAC, indicating that the mechanism of promoting longevity matches well with a prooxidant-mediated mitohormetic response [139]. Also, the involvement of miRNAs in the hormetic activation of the antioxidant system is suggested in an experiment of preharvest ultraviolet C (UV-C) treatment that attenuates postharvest senescence of stored strawberry fruit. Indeed, miR-159 and miR-398 are downregulated by UV-C, and that their respective targets are upregulated at the early stage of storage with enhancement of the activity of antioxidant enzymes. The initial burst of ROS primes the fruit in an antioxidative activated state via ROS-mediated feedback control with posttranscriptional involvement of miRNAs [140].

#### 3.4.3. Ischemic Preconditioning and miRNAs

Neuroprotective regulation of miRNAs is induced also in the early phase of ischemic preconditioning. MiR-200c, upregulated in the hippocampus of AD subjects [93] and in the cortices of APP transgenic mice at early period of A*β* deposition [113], is involved among miRNAs that upregulated at 3 hours after cerebral ischemic preconditioning in mice [141]. Of note, the protective function of miR-200c upregulation is mediated by targeting prolyl hydroxylase 2 (PHD2, also known as EGLN1), a sensor of oxygen and OS [142]. Similarly, downregulation of miR-15b, observed in the temporal cortex of AD patients [143], is associated with preconditioning by a volatile anesthetic sevoflurane against cerebral ischemic injury in rats by sustained expression of its target Bcl-2, an antiapoptotic protein [144].

## 4. MicroRNAs in Diagnosis and Therapy for Alzheimer's Disease

### 4.1. MiRNAs as a Diagnostic Tool for AD

#### 4.1.1. Circulating miRNAs in Bodily Fluids

MiRNAs are not only detected in tissue but also in bodily fluids such as blood plasma and serum, cerebrospinal fluid (CSF), urine, and saliva. These miRNAs are collectively known as circulating miRNAs. miRNAs are actively and selectively excreted from cell cytoplasm to bodily fluids through exosomes, microparticles, apoptotic cell bodies, or excreted as microvesicle free miRNAs that are associated with diverse proteins such as Ago and high-density lipoprotein. Also, they can be passively excreted into bodily fluids as a result of apoptosis, metastasis, and inflammation [90]. It has been suggested that miRNAs from the brain are able to cross the blood-brain barrier (BBB) into peripheral circulation using exosomes [145, 146].

#### 4.1.2. Circulating miRNAs in AD

According to a recent review on circulating miRNAs, collectively, 253 miRNAs have been reported with different expression levels in AD patients compared to healthy controls, of which 100 miRNAs are upregulated, 115 miRNAs are downregulated, and 38 miRNAs are both up- and downregulated in AD [90]. In another systematic review extracting dysregulated miRNAs in the peripheral blood from patients with AD, the authors have crossreferenced the dysregulated circulating miRNAs against the dysregulated miRNAs in the brain at the Braak stage III and identified 10 miRNAs [147]. Indeed, the list of 10 miRNAs are identical to those shown in Table 3, indicating that dysregulated miRNAs in association with both early-stage AD pathology and OS regulation can be detected in the peripheral blood. Four of these 10 miRNAs, namely, miR-107, miR-125b, miR-146a, and miR-34a, are found to be altered in the CSF in the same direction as observed in the brain [148, 149]. However, three miRNAs of the 10 miRNAs, namely, miR-125b, miR-146a, and miR-26b, are differently dysregulated between the brain and blood: upregulated in the brain but downregulated in the peripheral blood. This is probably due to the timing of sampling, since several miRNAs can be inversely dysregulated between early and late stage of AD. For example, miR-146a in the brain and CSF is upregulated at the Braak stage III of prodromal AD but downregulated at the Braak stage VI of advanced AD [94].

#### 4.1.3. Diagnostic Potential of Circulating miRNAs in AD

Of particular note, among the miRNAs shown in Table 3, plasma miR-107 and serum miR-125b measured with a real-time quantitative reverse transcriptase polymerase chain reaction (qRT–PCR) method has been found to show considerably high sensitivity (0.90 for miR-107 and 0.81 for miR-125b) and specificity (0.78 for miR-107 and 0.68 for miR-125b) as a biomarker of AD [149, 150]. Furthermore, decrease in plasma miR-107 is more prominent in amnestic MCI (aMCI) than in AD. Therefore, plasma miR-107 shows even higher sensitivity of 0.98 and specificity of 0.83 to discriminate between patients with aMCI and healthy controls, suggesting a potential role of the circulating miRNA as a diagnostic biomarker at the prodromal stage of AD [149]. Also, serum miR-34c is significantly increased in patients with aMCI and might be a predictive biomarker for diagnosis of aMCI [109].

### 4.2. MiRNAs as a Therapeutic Target of AD

#### 4.2.1. Recent Advances in RNA-Based Therapeutics

RNA-based therapeutics, such as small interfering RNAs (siRNAs), miRNAs, antisense oligonucleotides (ASOs), aptamers, synthetic mRNAs, and CRISPR-Cas9, have great potential to target currently undruggable genes and gene products and to generate new therapeutic paradigms in disease, ranging from cancer to pandemic influenza to AD [151]. Indeed, patisiran and givosiran are the U.S. Food and Drug Administration (*FDA*) approved, the first and second RNA interference- (RNAi-) based drugs indicated for the treatment of adults with polyneuropathy of hereditary transthyretin-mediated (hATTR) amyloidosis and acute hepatic porphyria (AHP), respectively [152, 153]. According to the recent systematic review, 28 ongoing studies are found to be related to the application of RNAs in the treatment and diagnosis of AD, i.e., ASOs (8 targets), miRNA mimics (agomirs) (7 targets), anti-miRNAs (antagomirs) (6 targets), siRNAs (5 targets), and mRNAs (2 targets) [154].

#### 4.2.2. Agomirs and Antagomirs as Therapeutics for AD

The field of miRNAs as therapeutics for AD has attracted attention and potentially offers a variety of solutions including A*β* or tau reduction, enhancement of neuronal survival, inhibition of apoptosis, and protection of synapses [155]. The therapeutic modulation of miRNAs can be done in two ways. The levels of downregulated or upregulated miRNAs can be reversed using agomirs or antagomirs which are antisense molecules that act to bind and thus inactivate the target miRNA sequence, respectively [156]. Specifically, an intraventricular infusion of miR-107 mimic (agomir) reverses the impairments of spatial memory and long-term potentiation and loss of pyramidal neurons caused by A*β* neurotoxicity in a mouse model of AD via the intraventricular injection of A*β*42 [157]. Conversely, the intrahippocampal delivery of a microRNA-146a specific inhibitor (antagomir) into transgenic mice with five familial AD mutations (5xFAD mice) showed enhanced hippocampal levels of rho-associated, coiled-coil containing protein kinase 1 (ROCK1) protein and repressed tau hyperphosphorylation, partly restoring the memory function in the 5xFAD mice [102]. Similarly, administration of miR-34c antagomir by the third ventricle injection or intranasal delivery markedly increased the brain levels of synaptotagmin 1 (SYT1) and ameliorated the cognitive function in SAMP8 mice [109]. Therefore, several OS-associated miRNAs summarized in Table 3 might be not only diagnostic but also therapeutic targets for AD.

## 5. Conclusion

Neuronal RNA oxidation in vulnerable neuronal populations in AD [54] and PD [158] was reported in 1999. Since then, involvement of RNA oxidation in the disease pathogenesis has been suggested in diverse neuropsychiatric disorders [4, 8] as well as common chronic diseases beyond the central nervous system such as diabetes and heart failure [159]. In 2015, the existence and the biological significance of oxidized miRNAs were demonstrated in cellular and animal models [19]. Cellular consequences of oxidatively altered RNAs have recently been investigated more intensively and rigorously than before, which provides still limited but an increasing amount of information about disrupted cellular functions in translational machinery and noncoding regulatory mechanisms. Indeed, three independent pathways of apoptosis induction have been identified through formations of oxidized mRNA [16], oxidized tRNA [68], and oxidized miRNA [19]. Besides direct oxidation, expression levels of several miRNAs embedded in upstream regulators or downstream targets of OS are altered in AD. It is noteworthy that both direct oxidation of RNAs and oxidative dysregulation of the miRNA expression occur at an early stage of neurodegeneration. Further understanding of the consequences and cellular handling mechanisms of the oxidatively altered RNAs may provide clues to the underlying mechanisms of neurodegenerative diseases and lead to better early intervention strategies.

## Figures and Tables

**Table 1:** Reasons why RNA species in human brain are vulnerable to oxidative insults.

RNA species	(i) Abundance in a cell (ii) Mostly single-stranded form and less protection by proteins (iii) Localization in the vicinity of mitochondria, the major source of ROS (iv) No known repair mechanisms for oxidized RNA
Human brain	(i) High oxygen consumption rate (ii) High content of easily peroxidizable polyunsaturated fatty acids (iii) High content of transition metals that catalyze ROS-generating reactions (iv) Low content of antioxidant enzyme catalase

ROS: reactive oxygen species.

**Table 2:** Oxidized RNA species potentially induce apoptosis.

RNA species	Mechanism inducing apoptosis by oxidized RNAs	Ref
Oxidized messenger RNA (mRNA)	Specific binding of poly(C)-binding protein 1 (PCB1) to heavily oxidized mRNA carrying two 8-oxo-guanine residues at the 9th and 15th positions triggers caspase-3 activation and subsequent apoptosis.	[16]
Oxidized transfer RNA (tRNA)	Because tRNA is accessible in the mitochondrial intermembrane space, oxidation of tRNA can be catalyzed by cytochrome c (cyt c) and leads to the formation of cross-linking complex between tRNA and cyt c. then, oxidized tRNA facilitates cyt c release from mitochondria and subsequently induces apoptosis.	[68]
Oxidized microRNA (miRNA)	Oxidized miRNA-184 containing 8-oxo-guanosine associates with the 3′ UTRs of Bcl-xL and Bcl-w that are not its native targets. Subsequent reduction in Bcl-xL and Bcl-w is responsible for the cells undergoing apoptosis.	[19]

3′ UTRs: three prime untranslated regions.

**Table 3:** Dysregulated microRNAs in association with both early-stage Alzheimer's disease (AD) pathology and oxidative stress (OS).

miRNA	Up- or downregulated in brains with AD pathology	Target gene	Specific function and association with OS regulation clarified by cellular and animal experiments	Ref
miR-107	Downregulated in the temporal cortex at BraaK stages III/IV of MCI subjects	BACE1	MiR-107 decreases BACE-1 mRNA levels by binding to the 3′ UTR of BACE1, hence decreases the production of A*β*. OS served as the key trigger to downregulate miR-107 by cell-derived soluble A*β*.	[92, 115, 116]
miR-125b	Upregulated in the frontal cortex at BraaK stages III/IV of non-demented and early AD subjects	SPHK1 NCAM	Overexpression of miR-125b promotes APP and BACE1 expression and A*β* production and apoptosis by targeting SPHK1 through inflammation (via increase in TNF-*α* and IL-6) and OS (via decrease in SOD). Mir-125b promotes tau phosphorylation by targeting NCAM.	[93, 99, 100]
miR-146a	Upregulated in the hippocampus at BraaK stages III/IV of preclinical or early AD subjects	CFH ROCK1 SOD2	MiRNA-146a is NF-*κ*B-sensitive and activates inflammation by targeting 3′ UTR of CFH, an important repressor of the inflammatory response of the brain. Overexpression of miR-146a induces tau phosphorylation by targeting ROCK1 via inhibition of PTEN. Mir-146a is upregulated by ROS and downregulates SOD2 (mitochondrial manganese SOD).	[94, 101–103]
miR-200c	Upregulated in the hippocampus at BraaK stages III/IV of non-demented and early AD subjects.	PTEN S6K1	In APP/PSEN1 double-transgenic mice, A*β* deposition results in ER stress that induces miR-200c. MiR-200c supports cell survival and neurite outgrowth of cultured neuron by targeting PTEN. MiR-200c reduces A*β* secretion by targeting S6K1 via reduction of IRS-1pSer and promoting insulin signaling. MiR-200c is upregulated by ROS in primary hippocampal neuron.	[93, 111–113]
miR-210	Downregulated in the hippocampus at BraaK stages III/IV of non-demented and early AD subjects	ISCU1/2 COX10	Soluble A*β* leads to NMDAR overactivation, excessive calcium influx, mitochondrion-derived ROS production, and upregulation of miR-210. MiR-210 targets ISCU1/2 and COX10 that have important roles in mitochondrial respiration and function	[93, 116]
miR-26b	Upregulated in the temporal cortex at BraaK stage III of patients with MCI	RB1	Overexpression of miR-26b leads to aberrant cell cycle re-entry and increased tau-phosphorylation by targeting RB1 via activation of RB1/E2F cell cycle and CDK5. Sequence-specific inhibition of miR-26b in culture is neuroprotective against OS.	[95]
miR-30e	Upregulated in the hippocampus at BraaK stages III/IV of non-demented and early AD subjects	SNAI1	Overexpression of miR-30e increases the levels of SOD, GSH, and GSH-PX and decreases ROS levels by targeting SNAI1 through decreasing TGF-*β* and SMAD2 expression and increasing NOX4 expression.	[93, 110]
miR-34a	Upregulated in the frontal cortex and hippocampus at BraaK stages III/IV of non-demented and early AD subjects.	SIRT1 ADAM10 NMDAR 2B TREM2 BCL2	Overexpression of miR-34a increases the levels of an adaptor protein p66shc and reduces tolerance to OS by targeting SIRT1. Conditional miR-34a overexpression mouse shows cognitive impairment associated with accumulation of intracellular A*β* and tau hyperphosphorylation possibly through targeting ADAM10, NMDAR 2B, and SIRT1. MiR-34a targets also the amyloid sensing- and clearance receptor protein TREM2 as well as anti-apoptotic protein BCL2.	[93, 104–108]
miR-34c	Upregulated in the hippocampus at BraaK stages III/IV of early AD subjects	SYT1	Overexpression of miR-34c mediates synaptic and memory deficits by targeting SYT1 through ROS generation, JNK activation, and p53 accumulation.	[94, 109]
miR-485	Downregulated in the frontal cortex at BraaK stage III of early AD subjects	BACE1 RAC1	MiR-485 decreases BACE1 mRNA levels by binding to BACE1 exon 6, hence decreases the production of A*β*. MiR-485 upregulation alleviates ischemia-reperfusion injury by targeting RAC1 via NOTCH2 signaling, which are evidenced by improved cell viability, decreased OS markers, and reduced apoptotic rate.	[96, 118, 119]

3′ UTR, three prime untranslated region; A*β*, amyloid-*β*; *ADAM10,* A Disintegrin and metalloproteinase domain-containing protein 10; APP, amyloid precursor protein; BACE1, *β*-site amyloid precursor protein-cleaving enzyme 1; BCL2, B-cell lymphoma 2; CDK5, cyclin-dependent kinase 5; CFH, complement factor H; *COX10*, cytochrome c oxidase assembly protein; ER, endoplasmic reticulum; GSH, glutathione; GSH-PX, glutathione-peroxidase; IL-6, interleukin-6; IRS-1pSer, insulin receptor substrate 1 at serine residues; ISCU1/2, iron-sulfur cluster scaffold homolog 1/2; *JNK*, Jun amino terminal kinase; MCI, mild cognitive impairment; NCAM, neural cell adhesion molecule; NF-*κ*B, nuclear transcription factor *κ*B; NMDAR, N-methyl-d-aspartate receptor; NOTCH2, neurogenic locus notch homolog protein 2; *NOX4*, NADPH oxidase 4; p66shc, 66 kDa proto-oncogene Src homologous-collagen homologue; PSEN1, presenilin-1; *PTEN*, *phosphatase and tensin homolog; RAC1*, RAS-related C3 botulinus toxin substrate 1; RB1, *retinoblastoma* 1; ROCK1, rho-associated, coiled-coil containing protein kinase 1; ROS, reactive oxygen species; S6K1, S6 kinase B1; SIRT1, *silent mating type information regulation 2 homolog* (sirtuin) 1; SMAD2, mothers against decapentaplegic homolog 2; *SNAI1*, snail family transcriptional repressor 1; SOD, superoxide dismutase; SPHK1, sphingosine kinase 1; *SYT1*, synaptotagmin 1; TGF, transforming growth factor; TNF-*α*, tumor necrosis factor-*α*; TREM2, triggering receptor expressed in myeloid cells 2.

## Additional Points

Since the submission of this article, it has been reported that formation of 8-oxo-guanine in seed region of miR-1 is sufficient to cause cardiac hypertrophy in mice, which provides novel evidence for an epitranscriptional role of oxidized miRNA in phenotypic alteration (H. Seok, H. Lee, S. Lee et al., “Position-specific oxidation of miR-1 encodes cardiac hypertrophy,” *Nature*, vol. 584, no. 7820, pp. 279-285, 2020).
